# Effect of clinical versus administrative data definitions on the epidemiology of *C. difficile* among hospitalized individuals with IBD: a population-based cohort study

**DOI:** 10.1186/s12876-022-02223-y

**Published:** 2022-03-26

**Authors:** Seth R. Shaffer, Zoann Nugent, Charles N. Bernstein, Andrew Walkty, Harminder Singh

**Affiliations:** 1 University of Manitoba IBD Clinical and Research Center, Winnipeg, MB Canada; 2 Internal Medicine, Section of Gastroenterology, University of Manitoba, 805-715 McDermot Avenue, Winnipeg, MB R3E3P4 Canada; 3 CancerCare Manitoba, Research Institute, Winnipeg, MB Canada; 4 Community Health Sciences, University of Manitoba, Winnipeg, MB Canada

**Keywords:** Case definition, *Clostridioides difficile*, Epidemiology, IBD

## Abstract

**Background:**

Hospitalization admissions and discharge databases (DAD) using the International Classification of Diseases (ICD) codes are often used to describe the epidemiology of *Clostridioides difficile* infections (CDI) among those with Inflammatory bowel disease (IBD), even though DAD CDI definition can miss many cases of CDI. There are no data comparing the assessment of the epidemiology of CDI among those with IBD by DAD versus laboratory diagnosis. We used a population-based dataset to determine the effect of using DAD versus laboratory CDI diagnosis on CDI assessment among those with IBD.

**Methods:**

We linked the University of Manitoba IBD Epidemiology Database to the provincial CDI laboratory dataset for the years 2005–2014. Time trends of CDI were assessed using joinpoint analyses. We used stratified logistic regression analysis to assess factors associated with CDI among individuals with IBD.

**Results:**

Time trends of CDI among hospitalized individuals with IBD were similar when using DAD or the laboratory CDI diagnosis. Prior hospital admission and antibiotic exposure were associated with CDI using either of the CDI definitions, 5-ASA use was associated with CDI using DAD but not laboratory diagnosis, whereas corticosteroid exposure was associated with laboratory-based CDI diagnosis. Using laboratory results as gold standard, DAD had a sensitivity and specificity of 75.4% and 99.6% for CDI among those with IBD.

**Conclusions:**

Using ICD codes in the DAD for CDI provides similar epidemiological time trend patterns as identifying CDI in the laboratory dataset. Hence, ICD codes are reliable to determine CDI epidemiology among hospitalized individuals with IBD.

**Supplementary Information:**

The online version contains supplementary material available at 10.1186/s12876-022-02223-y.

## Background

*Clostridioides difficile* (*C. difficile*) is the most frequently reported nosocomial pathogen [1], and is responsible for up to 30% of antibiotic associated diarrhea [2]. Among patients with inflammatory bowel disease (IBD), *C. difficile* infections (CDI) are associated with worse clinical outcomes, including increased emergency room visits, longer hospitalizations, higher rates of colectomy, and increased mortality rates [3–5]. Infection with CDI may mimic an IBD flare and CDI may precipitate an IBD flare. Therefore, treatment of an individual with IBD with active CDI can be difficult, as it involves treating both CDI with antibiotics, and optimal management of the patient’s immunosuppression [3]. We have previously shown in a population-based cohort that individuals with IBD have a 4.8-fold increased risk of laboratory confirmed CDI compared to people without IBD, with no difference in rates between those with ulcerative colitis (UC) or Crohn’s disease (CD) [6].

Much of the information on the epidemiology of CDI among individuals with IBD in North America has traditionally come from an assessment of hospitalization admission and discharge databases (DAD), using the International Classification of Diseases (ICD), 9^th^ revision, Clinical Modification (ICD-9-CM) code for CDI [7, 8]. Using DAD can save time by removing the need to contact multiple diagnostic laboratories to obtain their data. Conversely, the accuracy of this method to identify CDI in the general population is highly variable [9, 10]. The introduction of ICD, 10^th^ revision (ICD-10) coding in Canada over the last decade, a more specific coding system that may lead to improvements in assessments using DAD.

We have previously reported that DAD have a limited accuracy in identifying the occurrence of CDI using ICD-10 CDI codes among hospitalized individuals with IBD, with approximately 30% of laboratory confirmed cases not identified in DAD [11]. However, it remains unknown whether the assessment of CDI epidemiology over time among hospitalized individuals with IBD, provides different patterns and risk factors when the laboratory dataset is used compared with using hospital discharges CDI code. It is possible there is no effect on the determination of time trends and epidemiology of CDI among those with IBD versus those without IBD, as perhaps there is no differential effect across study years.

Hence, the aim of our current study was to compare the epidemiological patterns of CDI among hospitalized individuals with IBD as assessed using ICD-10 CDI codes in the DAD to that from laboratory-confirmed CDI diagnosis in the same population-based setting.

## Methods

Manitoba is a central Canadian province with a population of 1.36 million people in 2018 [12]. Manitoba Healthy Seniors and Active Living (MHSAL) is the provincial agency which oversees the delivery of universal healthcare in the province. MHSAL maintains several electronic administrative healthcare databases to monitor the healthcare services delivered and for re-imbursement to health care providers for the services rendered. These include hospitalization admission and discharge database (DAD), physicians claims dataset (inpatient and outpatient physician visits), the Drug Programs Information Network (DPIN) database (all out patient prescription dispensations) and the provincial population registry (all permanent residents of Manitoba). Up to 16 ICD-9 (prior to 2004) or 25 ICD-10 (since 2004) diagnostic codes are recorded for each inpatient hospital stay, whereas each outpatient physician visit is coded with a single ICD-9 diagnostic code.

The University of Manitoba IBD Epidemiology Database (UMIBDED) was initiated in 1995 and is repeatedly updated using MHSAL administrative databases and therefore contains all of the information listed above for MHSAL administrative databases [13]. The case definition of IBD in UMIBDED includes individuals with at least 5 separate physician contacts and/or hospitalizations for an IBD diagnosis (≥ 3 contacts for those residing in Manitoba for ≤ 2 years). This case definition has been previously validated, with a sensitivity and specificity of approximately 90% in comparison with both patient self-report and chart review [13]. The UMIBDED has been used for many epidemiological studies [14–18]. Cases of IBD in the UMIBDED, (as of March 2015, n = 8,277), are matched 1:10 to age, sex and area of residence (at time of IBD diagnosis) matched controls (n = 72,387). We include patients with J-pouches, ostomies, or indeterminate colitis.

The MHSAL Public Health Branch Epidemiology and Surveillance Unit has maintained a population-based CDI database since 2005**,** developed from the legally mandated universal reporting of documented CDI cases in the province to the unit. Diagnostic testing for CDI is performed by five public laboratories, of which four perform most (approximately 99%) of the testing. Only loose stool, which takes the shape of its container, is tested by the laboratories, thereby minimizing the detection of asymptomatic carriers (estimated to be 2–7% of the population [19, 20]). Between April 2005 and May 2013, Manitoba laboratories performed immunoassays for the glutamate dehydrogenase (GD) antigen and *C. difficile* toxins A and B, followed by the cytopathic effect (CPE) assay (using viable human fibroblasts) and/or culture for discordant results (i.e., GD antigen positive *C. difficile* toxin A & B immunoassay negative) [21, 22]. Since May 2013, three laboratories (responsible for approximately 70% of the testing) implemented a Nucleic Acid Amplification Test (the Illumigene assay, Meridian Biosciences, Cincinnati, OH) for confirmation of GD antigen positive samples [22]. The testing strategies used are among those recommended by the Infectious Diseases Society of America Guidelines for *C. difficile* Infection.

All residents of the province have been assigned a personal health identification number (PHIN) since 1984. PHIN is used to link the data in the databases.

### Study measures

The UMIBDED (April 1, 1984–March 31, 2014) was used to identify individuals with IBD, who were matched 1: 10 to those without IBD on age, sex, and area of residence (at IBD diagnosis/ index date). Everyone was followed individually longitudinally. Since DAD can only determine CDI among hospitalized patients, we restricted our analysis to that of CDI among hospitalized patients.

The DAD CDI diagnosis (A04.7) was determined among those hospitalized between July 1, 2005–March 31, 2014. The Manitoba Health Public Health Branch Epidemiology and Surveillance population-based CDI dataset was used to identify and define laboratory confirmed CDI cases (July 1, 2005 to March 31, 2014).

Each individual with IBD with CDI diagnosis during a hospitalization was matched with four IBD cases without a hospital record of CDI on or prior to the CDI index date (hospital admission date for first hospitalization with CDI), according to IBD type (UC vs. CD), gender, year of IBD diagnosis, and age.

Medication use (5-ASA, thiopurine, anti-TNF, corticosteroids) among those with IBD was defined as out-patient dispensation of one or more prescriptions in the year prior to the index hospitalization with CDI. Antibiotic use, including ciprofloxacin, metronidazole, and clindamycin, were defined as out-patient dispensation of one or more prescriptions in the three months prior to developing CDI. Hospital admission was defined as at least one overnight admission to the hospital in the year prior to developing CDI, and surgery was defined as at least one surgical procedure (involving complete or partial excision of the small or large bowel) at any time prior to developing CDI. Comorbidity burden was determined from ambulatory care and hospital admission diagnoses in the one year prior to CDI index date using the Charlson Comorbidity Index (CCI) score [23]. Frequency of ambulatory care visits in the year prior to developing to CDI was categorized into quartiles, 0–8 visits comprising the first quartile, 9–16 visits the second, 17–27 the third quartile, and 28 + the fourth.

### Statistical analyses

Joinpoint Regression program [24] developed by SEER was used to assess the time trends of CDI rates. Annual percent change (APC) and 95% confidence intervals (95% CI) were calculated.

We assessed factors associated with CDI among hospitalized individuals with IBD using CDI DAD code and compared with the factors determined using laboratory dataset CDI diagnosis to define CDI. Stratified logistic regression was used to determine predictors of CDI among hospitalized individuals with IBD, whether CDI was identified by laboratory dataset or DAD with strata being the case with CDI and associated control set. Variables from the univariable analysis with a *p*-value < 0.10 were used in the multi-variable analysis to identify factors associated with CDI among hospitalized individuals with CDI.

Sensitivity, specificity, predictive values and diagnostic values were compared using Fisher’s exact test. Data from the July 2005 to May 2013 time period were compared to May 2013 to March 2014 to assess if the laboratory change in CDI diagnosis in May 2013 affected the test characteristics results. We used Dice similarity coefficient to compare CDI identified by the two definitions among those with IBD. Dice similarity coefficient, also called proportion of specific agreement, measures overlap between two groups. A good overlap occurs at a level greater than 0.70 [25, 26].

Statistical significance was determined at a *p*-value of less than 0.05. Statistical analyses were carried out using SAS 9.4 software (SAS Institute, Cary, NC). This study was approved by the University of Manitoba’s Health Research Ethics Board and MHSAL’s Health Information and Privacy Committee.

## Results

A total of 64,692 hospital admissions were identified in the UMIBDED. Admissions after IBD diagnosis (N = 59,024) included 12,147 admissions among 4,244 IBD cases and 46,877 admissions among 22,285 individuals without IBD. There were 464 episodes of CDI after the IBD diagnosis/index date (matching date for those without IBD) among the study cohorts, using either of the laboratory dataset or DAD definition (CD:82; UC:85; individuals without IBD:297). Of the 383 patients with hospitalization for CDI, 326 had one CDI episode-196 had CDI by the laboratory dataset and DAD definition, 82 by laboratory dataset alone (DAD negative), and 48 by DAD definition only.

**Fig. 1:**
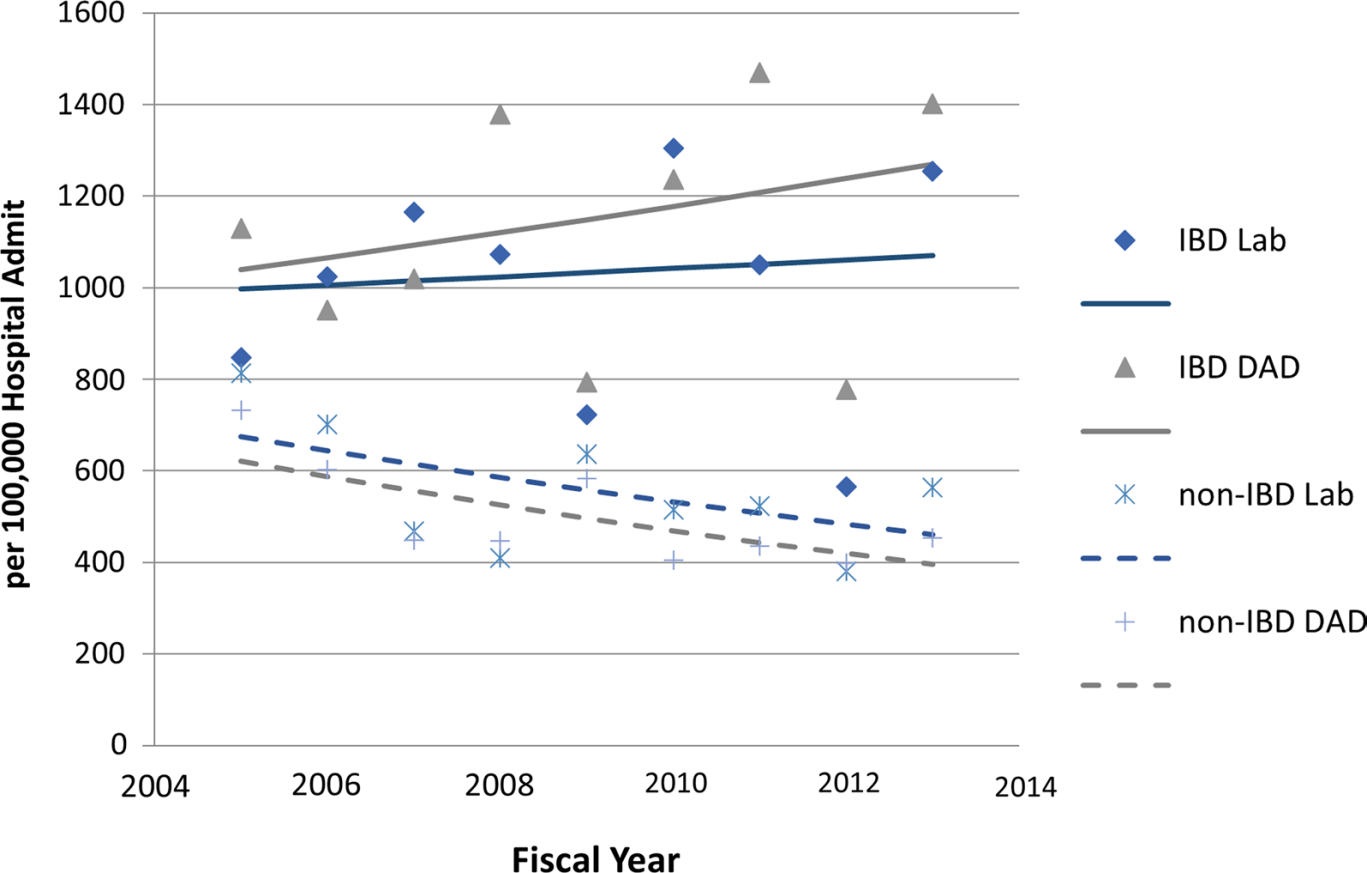
Annual CDI infection rates in IBD and those without IBD according to laboratory dataset (Lab) and Hospitalization Admission and Discharge Database (DAD) (A047). Annual percentage change and *p* values: IBD Lab: 0.89, 0.81; IBD DAD: 2.53,0.45; Non-IBD Lab: − 4.66, 0.12; Non-IBD Lab − 5.48, 0.02

**Fig. 2:**
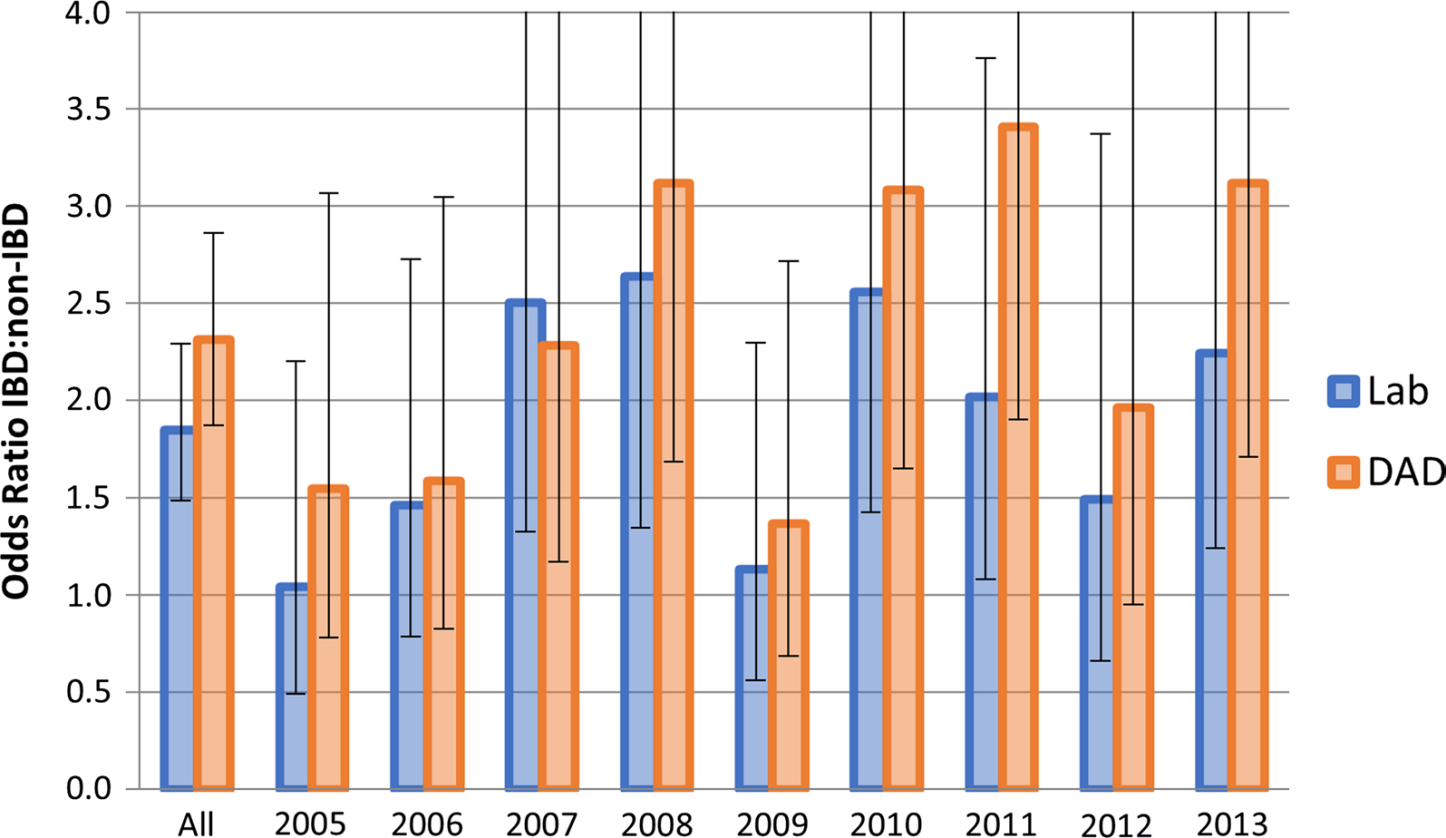
Odds ratio of CDI in IBD compared to those without IBD using laboratory dataset (Lab) and hospitalization admission and discharge database (DAD) (A047)

Among hospitalized individuals with IBD (Fig. 1), CDI assessment from DAD as well as the laboratory dataset showed no increase in CDI rates between 2005 and 2014, (DAD APC =  + 2.5%, 95% CI − 4.8, 10.4, *p* = 0.45; laboratory dataset APC =  + 0.9%, 95% CI − 7.0, 9.5, *p* = 0.80). In contrast, there was a statistically significant decrease in the CDIs among individuals without IBD when using laboratory dataset (APC = − 5.5%, 95% CI − 9.8, − 0.9, *p* = 0.025), and a statistically non-significant, but strong tendency of decrease based on the DAD (APC = − 4.7%, 95% CI − 10.6, 1.7, *p* = 0.12). These results lead to a slightly higher rate ratio of CDI in IBD to controls when using the DAD (OR 2.31) to compare the two groups (paired t-test *p* = 0.013), than when using laboratory dataset (OR 1.85) (Fig. 2). There was no consistent relationship for the odds ratios determined using the two different definition of CDIs (Additional file 1: Table S1). Dice similarity coefficient was 0.75, (95% CI 0.70–0.80) for IBD with CDI by the two definitions.

**Fig. 3:**
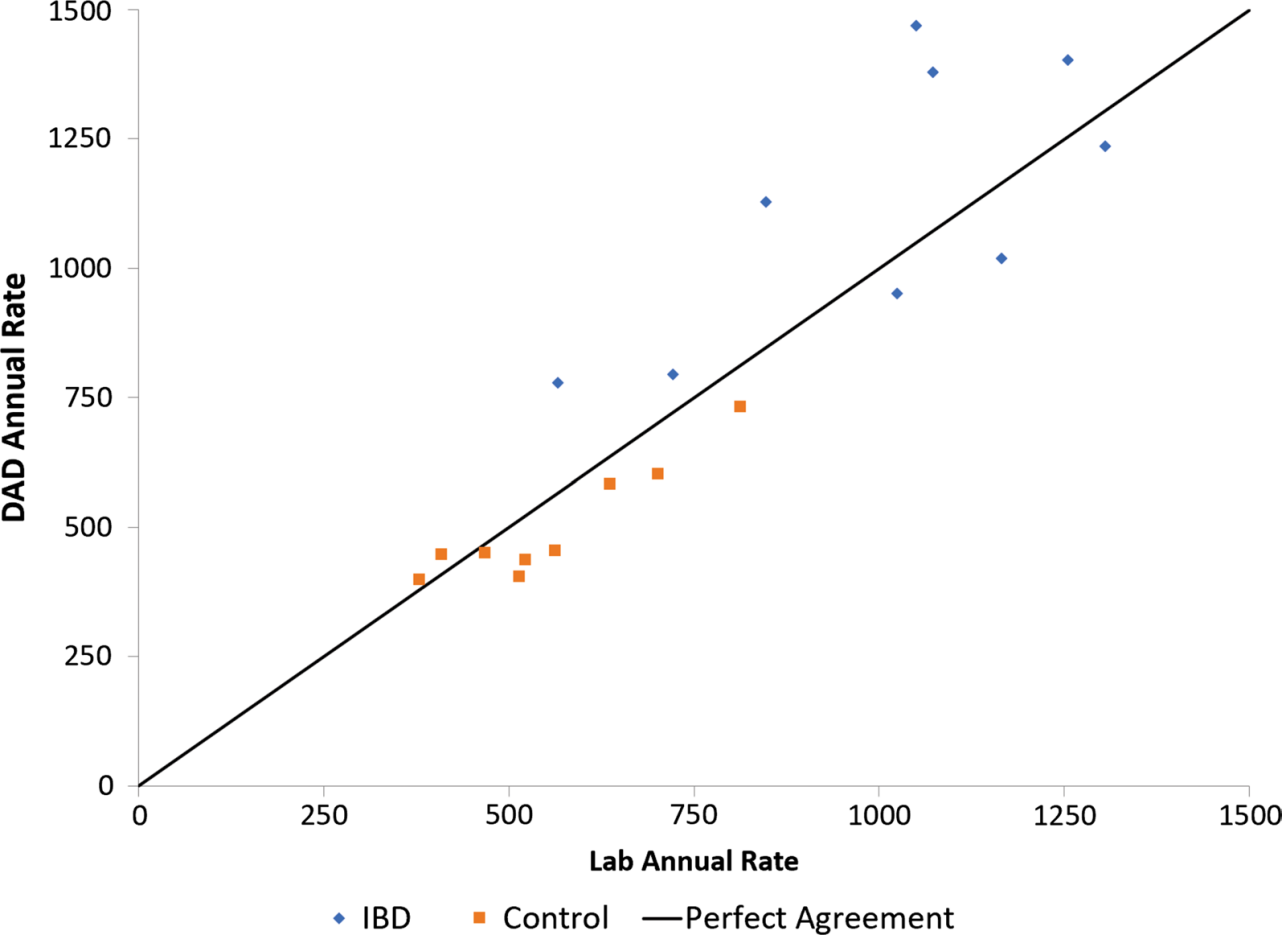
Correlation of CDI in the Laboratory dataset and Hospitalization Admission and Discharge Database (DAD) CDI diagnosis through the study years

Laboratory dataset and DAD CDI diagnosis were positively correlated with each other through the years (r = 0.72; *p* = 0.03; Fig. 3).

### Factors associated with CDI among hospitalized individuals with IBD

There were 108 individuals with IBD with CDI during hospitalizations identified by laboratory dataset, and 531 matched individuals with IBD without CDI (controls for this analysis) (Table 1). Similarly, there were 110 inpatients with IBD and CDI identified by DAD, with 539 matched individuals with IBD without CDI. Using laboratory dataset to determine IBD with CDI, 44% were male and median age at CDI diagnosis was 55.5, while using DAD 47% were male and the median age at CDI diagnosis was 60. The difference between IBD patient characteristics among those with and without CDI were similar when the two definitions of CDI were used (Table 1). There were no differences in the characteristics of individuals with CDI identified by laboratory dataset versus DAD either among those with or without IBD (Table 2).

**Table 1:** Comparison of IBD patients with and without CDI identified by laboratory dataset and by hospitalization admission and discharge database (DAD)

	Laboratory dataset			DAD		
	IBD patients with CDI (n = 108)	Matched IBD patients without CDI (n = 531)	*p*-value	IBD patients with CDI (n = 110)	Matched IBD patients without CDI (n = 539)	*p*-value
Age median (IQR)	55.5(34–75)	55 (34–75)		60 (40–75)	59 (40–74)	
% CD	51	51		46	47	
% Male	44	44		47	48	
Use 1 year prior						
5-ASA use (%)	47.2	37.9	0.084	51.8	39.2	**0.015**
Thiopurine use (%)	19.4	16.4	0.48	16.4	13.5	0.45
Anti-TNF use (%)	7.4	8.7	0.85	10.9	8.0	0.35
Corticosteroid use (%)	45.4	22.6	**< 0.001**	40.9	22.3	** < 0.001**
Use 3 month prior						
Any antibiotic use (%)	50.0	23.5	**< 0.001**	50.9	19.8	** < 0.001**
Ciprofloxacin use (%)	16.7	6.4	**0.002**	16.4	5.2	** < 0.001**
Metronidazole use (%)	17.6	4.7	**< 0.001**	16.4	3.3	** < 0.001**
Clindamycin use (%)	Data too sparse to report N < 6		**0.031**	Data too sparse to report N < 6		**0.016**
Hospitalization in the prior year (%)	62.0	31.1	**< 0.001**	63.6	31.5	**< 0.001**
Surgery (%)	23.2	23.9	0.90	20.0	25.1	0.28
Charlson Comorbidity index score (%)			**0.002**			**0.004**
0	49.1	61.6		42.7	59.0	
1	16.7	19.4		20.0	19.1	
2	21.3	9.6		17.3	9.5	
3+	13.0	9.4		20.0	12.4	
Ambulatory care (%)			**< 0.001**			**< 0.001**
Lowest quartile	11.1	29.8		10.0	30.2	
2nd quartile	15.7	25.8		14.6	25.1	
3rd quartile	34.3	22.2		34.6	23.4	
Highest quartile	38.9	22.2		40.9	21.3	

^a^Ambulatory care frequency quartiles: Quartile 1 0–8, Quartile 2 9–16, Quartile 3 17–27, Quartile 4 28+

*Bolded *p*-values are statistically significant

**Table 2:** Comparison of individuals with CDI identified by laboratory dataset versus hospitalization admission and discharge database (DAD) among those with and without IBD

	IBD			Individuals without IBD		
	CDI identified by laboratory dataset	CDI identified by DAD	*p* value	CDI identified by laboratory dataset	CDI identified by DAD	*p* value
	(n = 108)	(n = 110)		(n = 226)	(n = 201)	
Age Median (IQR)	55.5(34–75)	60 (40–75)	0.49	74.5 (63–82)	72 (61–82)	0.38
% CD	51	46	0.59			
% Male	44	47	0.59	43	42	0.77
Use 1 year prior						
5-ASA use (%)	47.2	51.8	0.50			
Thiopurine use (%)	19.4	16.4	0.60			
Anti-TNF use (%)	7.4	10.9	0.48			
Corticosteroid use (%)	45.4	40.9	0.58			
Use 3 month prior						
Any antibiotic use (%)	50.0	50.9	1	50.0	49.2	0.92
Ciprofloxacin use (%)	16.7	16.4	1	19.9	17.9	0.62
Metronidazole use (%)	17.6	16.4	0.86	8.0	9.0	0.73
Clindamycin use (%)	Data too sparse to report N < 6	Data too sparse to report N < 6	1	6.6	7.0	1
Hospitalization in the prior year (%)	62	63.6	0.89	56.2	55.7	0.92
Surgery (%)	23.2	20	0.62	Data too sparse to report N < 6	3.0	0.76
Charlson comorbidity index score (%)			0.41			0.90
0	49.1	42.7		22.6	19.9	
1	16.7	20		18.1	19.4	
2	21.3	17.3		18.6	17.9	
3+	13	20		40.7	42.8	
Ambulatory care (%)			0.98			0.87
Lowest quartile	11.1	10		15.5	15.9	
2nd quartile	15.7	14.6		25.2	23.9	
3rd quartile	34.3	34.6		21.7	24.9	
Highest quartile	38.9	40.9		37.6	35.3	

^a^Ambulatory care frequency quartiles: Quartile 1 0–8, Quartile 2 9–16, Quartile 3 17–27, Quartile 4 28 +

In univariable analysis, CDI identified by laboratory dataset or by DAD, was associated with exposure to corticosteroids, any antibiotic, metronidazole, ciprofloxacin, and clindamycin (Table 3). Exposure to 5-ASA was associated with CDI identified by DAD, but not with CDI in the laboratory dataset. Increased ambulatory care use, hospitalization in the previous year, and greater comorbidity was associated with CDI identified by either DAD or laboratory dataset.

**Table 3:** Univariable analysis of factors associated with CDI in hospitalized IBD patients according to CDI defined by the laboratory dataset or hospitalization admission and discharge database (DAD)

	Laboratory dataset		DAD	
	Odds ratio (95% CI)	*p*-value	Odds ratio (95% CI)	*p*-value
5-ASA	1.53 (0.99–2.37)	0.054	1.75 (1.14–2.68)	**0.010**
Thiopurines	1.30 (0.74–2.30)	0.36	1.33 (0.73–2.43)	0.36
Anti-TNF	0.85 (0.38–1.91)	0.69	1.51 (0.73–3.11)	0.26
Corticosteroids	2.81 (1.83–4.32)	**< 0.001**	2.45 (1.59–3.78)	**< 0.001**
Any antibiotic	3.26 (2.10–5.05)	**< 0.001**	4.36 (2.78–6.83)	**< 0.001**
Metronidazole	4.24 (2.20–8.17)	**< 0.001**	5.51 (2.72–11.2)	**< 0.001**
Ciprofloxacin	2.95 (1.58–5.50)	**< 0.001**	3.73 (1.95–7.16)	**< 0.001**
Clindamycin	5.92 (1.30–26.9)	**0.021**	5.70 (1.51–21.5)	**0.010**
Prior hospital admission	3.48 (2.28–5.33)	**< 0.001**	3.81 (2.46–5.89)	**< 0.001**
Surgery	0.97 (0.58–1.62)	0.90	0.74 (0.44–1.25)	0.26
Charlson Comorbidity index score				
0	Reference		Reference	
1	1.20 (0.65–2.22)	0.57	1.64 (0.91–2.97)	0.10
2	3.18 (1.69–5.98)	**< 0.001**	2.90 (1.50–5.62)	**0.002**
3+	1.87 (0.94–3.75)	0.076	2.57 (1.39–4.74)	**0.003**
Ambulatory care				
Lowest quartile	Reference		Reference	
2nd quartile	1.82 (0.83–3.99)	0.14	1.88 (0.83–4.27)	0.13
3rd quartile	4.73 (2.31–9.70)	**< 0.001**	4.84 (2.36–9.94)	**< 0.001**
Highest quartile	5.45 (2.68–11.1)	**< 0.001**	6.69 (3.24–13.8)	**< 0.001**

^a^Ambulatory care frequency quartiles: Quartile 1 0–8, Quartile 2 9–16, Quartile 3 17–27, Quartile 4 28 +

*Bolded *p*-values are statistically significant

In the multi-variable analysis (Table 4), prior hospital admission and antibiotic exposure were associated with CDI using either of the CDI definitions, whereas corticosteroids were associated with CDI in the laboratory set, and 5-ASA exposure in the DAD.

**Table 4:** Multivariate analysis of factors associated with CDI in Hospitalized IBD patients according to CDI defined by lab dataset or hospitalization admission and discharge database (DAD)

	Lab dataset		DAD	
	Odds ratio (95% CI)	*p*-value	Odds Ratio (95% CI)	*p*-value
5-ASA	1.30 (0.81–2.10)	0.28	1.65 (1.02–2.65)	**0.040**
Corticosteroids	1.71 (1.05–2.79)	**0.031**	1.37 (0.82–2.28)	0.23
Any antibiotic	2.21 (1.37–3.56)	**0.001**	2.78 (1.70–4.54)	** < 0.001**
Prior hospital admission	2.00 (1.18–3.39)	**0.010**	2.24 (1.32–3.79)	**0.003**
Charlson Comorbidity index Score				
0	Reference		Reference	
1	0.67 (0.34–1.34)	0.26	1.01 (0.52–1.95)	0.98
2	1.72 (0.82–3.58)	0.15	1.45 (0.67–3.14)	0.34
3 +	0.83 (0.35–1.94)	0.66	0.91 (0.42–1.96)	0.80
Ambulatory care				
Lowest quartile	Reference		Reference	
2^nd^ quartile	1.25 (0.55–2.84)	0.60	1.19 (0.50–2.83)	0.69
3^rd^ quartile	2.29 (1.03–5.08)	**0.042**	2.14 (0.95–4.81)	0.067
Highest quartile	2.07 (0.85–5.04)	0.11	2.34 (0.96–5.72)	0.063

^a^Ambulatory care frequency quartiles: Quartile 1 0–8, Quartile 2 9–16, Quartile 3 17–27, Quartile 4 28 +

^*^Bolded *p*-values are statistically significant

Using laboratory results as the gold standard, DAD had a sensitivity and specificity of 75.4% and 99.6% for CDI among those with IBD, and 73.8% and 99.9% for CDI among those without IBD, respectively (Table 5). However, the PPV and NPV were both lower for CDI in DAD for those with IBD than among those without IBD. There was no statistical difference in test characteristics when separating IBD into CD or UC patients.

**Table 5:** Test characteristics of CDI in the Hospitalization Admission and Discharge Database (DAD) to CDI in the laboratory dataset

	IBD	No IBD	*p*-value	CD	UC	*p*-value
Admissions	12,147	46,877		6,792	5,355	
True positive	0.76	0.40	**< 0.0001**	0.63	0.92	0.18
False negative	0.25	0.14		0.27	0.22	
False positive	0.37	0.09		0.31	0.45	
True negative	98.63	99.37		98.79	98.41	
Overall diagnostic accuracy	99.38	99.77	**< 0.001**	99.43	99.33	0.56
Sensitivity	75.41	73.83	0.80	70.49	80.33	0.29
Specificity	99.63	99.91	**< 0.001**	99.69	99.55	0.23
Positive predictive value	67.15	82.17	**0.0014**	67.19	67.12	1
Negative predictive value	99.75	99.86	**0.016**	99.73	99.77	0.72

*UC* ulcerative colitis, *CD* Crohn’s disease

*Bolded *p*-values are statistically significant

Due to the laboratory differences in CDI diagnostic algorithm after May 2013, we repeated the analyses comparing DAD CDI definition to the laboratory CDI data stratified by time periods (July 2005 to May 2013 and May 2013 to March 2014). There were no significant differences in the true positive, true negative, false positive, false negative rates, sensitivity, and specificity between the two time periods.

## Conclusion

We are reporting that hospital discharge database, using ICD codes for CDI, provides very similar epidemiological time trends of CDI for the IBD population, when compared with CDI identified by laboratory confirmed data. CDI assessment using hospital discharge abstracts or laboratory dataset, both did not show an increase in CDI among hospitalized IBD patients. The CDI rate decreased for both laboratory dataset and DAD when assessing patients without IBD, with the laboratory dataset rate decrease being statistically significant. The factors associated with CDI were similar using either definition, except for association with use of corticosteroids or 5-ASAs. These patterns are evident even though there is lower PPV and NPV of the hospital discharge database CDI code for CDI among those with IBD than among those without IBD as compared to laboratory CDI diagnosis.

While results based on DAD have obvious shortcomings, such as missed CDI diagnoses, this did not affect the trends we saw over time. It did, however, affect the differences in CDI rates between patients with IBD compared to those without IBD, leading to a statistically significant higher rate ratio when using DAD to compare the two groups, compared with laboratory dataset, although the differences were very small and unlikely to be of clinical significance.

One issue of contention in clinical practice is the differentiation between active *C. difficile* infection and colonization. Stool samples in our province are only tested for *C. difficile* infection if the stool sample is liquid, and formed stool is therefore not tested for *C. difficile* to reduce the chance of picking up colonization. While the distinction between colonization and active *C. difficile* infection remains difficult among individuals with diarrhea, a positive *C. difficile* stool test in a person with pre-existing IBD is treated [27]. Further research is needed to develop better methods to distinguish *C. difficile* colonisation from active infection especially among those with IBD with diarrhea. This is an important ongoing problem with CDI research.

We have previously shown that thirty percent of laboratory confirmed CDI samples from inpatients are not recorded in hospital discharge abstracts and, the positive predictive value of ICD-10 CDI codes in hospital discharge databases is lower among those with IBD compared to persons without IBD [11]. Our current analysis confirms this finding. When using the laboratory dataset as the gold standard, the positive predictive value of DAD is lower for hospitalized IBD patients with CDI compared to those without IBD hospitalized with CDI. Therefore, while DAD is able to give similar epidemiological trends as the laboratory dataset, it does not accurately reflect the true numbers of hospitalized IBD patients with CDI. More importantly, DAD is unable to differentiate between community-acquired (before the patient is hospitalized) and hospital-acquired (patient acquiring while in hospital) CDI, which allows deciphering the significance of CDI in the community and within the hospital settings. Using the hospital dataset alone, it is impossible to assess for individuals acquiring CDI in hospital but manifesting with CDI after discharge.

Using the laboratory dataset as the gold standard, we reported sensitivity, specificity, positive and negative predictive values of using DAD as 75.4%, 99.6%, 67.15%, and 99.75%, respectively among those with IBD. A study in Calgary, Canada has previously reported sensitivity, specificity, positive predictive value, and negative predictive values of 82.1%, 99.4%, 88.4%, and 99.1%, respectively, for the performance of ICD-10 CDI codes among patients with ulcerative colitis [28]. The Calgary study did not assess patients without IBD, however, and hence could not compare the effect of using ICD-10 CDI codes on outcome differences between the two groups; neither did that study compare any differences in epidemiology using varied definitions. A study out of France reported an underestimation of CDI when using ICD-10 CDI codes among the general population [29].

Factors associated with CDI in the hospitalized IBD population using the laboratory dataset or DAD were similar in our study. In the multivariable analysis, exposure to corticosteroids was an independent factor associated with laboratory-based CDI diagnosis among hospitalized individuals with IBD. This association with corticosteroid use may be explained by clinicians treating a presumed IBD flare before testing for the presence of CDI in stool. Alternatively, this could be reflective of increased risk of CDI among those with IBD with severe disease, requiring corticosteroids; however anti-TNF use did not have a similar effect. It is also possible corticosteroids could increase risk of hospitalization with CDI. Rodemann et al. [30] assessed hospitalized IBD and non-IBD patients using hospital discharge codes and laboratory data, and found that the incidence of CDI was higher in all IBD patients (including UC and CD), and UC, though not CD, compared to non-IBD patients. Using multivariable logistic regression, IBD, older age (> 45), and Charlson comorbidity index were independent factors associated with positive *C. difficile* stool test. However, no prior study has assessed the effects of medication use prior to hospitalization on laboratory-based CDI diagnosis among hospitalized individuals with IBD.

In our study, antibiotic exposure in the prior three months, including ciprofloxacin, metronidazole, and clindamycin were associated with CDI defined by both DAD and the laboratory dataset, likely as antibiotics are a known risk factor for developing CDI [31]. It is also possible that antibiotics were used empirically to treat an acute diarrheal illness, despite guidelines recommending against this [32]. An increased number of co-morbidities, any hospital admission in the prior year and higher ambulatory care use in the prior year were also associated with hospitalization with CDI among those with IBD, suggesting such individuals should be aggressively assessed for CDI. This agrees with prior literature listing co-morbidities as well as increasing healthcare exposure as risk factors for developing CDI [30, 31].

Our paper’s strengths include using population-based databases, allowing us to include in our study all persons who receive care in the province of Manitoba. Further, because of using these databases, there was no referral bias, or recall bias, associated with our study. It is also potentially generalizable to other patient populations, as we have captured CDI rates in a population-based setting.

Our paper also has limitations, as we were unable to conduct a chart review, and acquire further clinical or laboratory data pertaining to patients’ clinical course. We were unable to differentiate between ileal and colonic Crohn’s disease; those with colonic disease have a higher risk of CDI compared to those with isolated small bowel disease [33]. Laboratory testing for *C. difficile* did change near the end of our study period, however, this did not affect comparison of CDI definition by laboratory dataset to that by DAD. Stool testing for *C. difficile* should be performed for every patient with diarrhea, but this may not always occur in clinical practice, and it’s possible some cases of CDI were missed and therefore not included in our datasets. As previously mentioned, it is difficult to distinguish between active *C. difficile* infection, and colonization, though it is current practice to treat *C. difficile* in a person with IBD with diarrhea, [27].

This is an observational study and there could be residual confounding in assessing factors associated with CDI among those with IBD; however, this should not be different by DAD versus laboratory definition of CDI. Then number of CDI episodes each year among those with IBD were low; however, we used joinpoint program which is designed to detect change in time trends of relatively rare events. Follow-up in our study ended in May 2014; additional studies would need to evaluate more recent practices.

In conclusion, our study suggests that results of studies using ICD codes to study the epidemiology of *Clostridioides difficile* infections may give similar epidemiological trends as using laboratory datasets and therefore ICD coding can be used when the laboratory dataset are not available. ICD codes have a very good specificity in detecting CDI with a poorer sensitivity, which will give erroneous raw numbers of CDI in the population and not allow evaluating individual cases with CDI. Therefore, while DAD may be used when assessing CDI time trends in persons with IBD, laboratory datasets are more accurate in understanding the true impact CDI has in the IBD population. Understanding epidemiologic trends and obtaining accurate numbers of CDI in those with IBD is integral to recognizing its impact in this population, as these patients have worse outcomes than those without CDI.

## Supplementary Information

**Additional file 1: Supplementary table 1**. Odds ratio of CDI in IBD compared to those without IBD using laboratory dataset (Lab) and Hospitalization Admission and Discharge Database (DAD) (A047).

## Data Availability

We would like to note that the data used in this analysis are owned by the government of Manitoba. We were given permission to use the data to conduct the analysis. However we do not have permission to share the data. Researchers interested in replicating our results, can apply to the ministry of health to access the data: Health Information Privacy Committee, Manitoba Health, Seniors and Active Living, 4043-300 Carlton Street, Winnipeg MB R3B 3M9 (email: hipc@gov.mb.ca). Instructions can be found at: http://www.gov.mb.ca/health/hipc/submission.html.
