# Supplementary material for: Effect of clinical versus administrative data definitions on the epidemiology of C. difficile among hospitalized individuals with IBD: a population-based cohort study
Source: BMC Gastroenterol. 2022 Mar 26;22:140. doi: 10.1186/s12876-022-02223-y (PMC8962161; doi:10.1186/s12876-022-02223-y)
Supplement: Supplementary file 1 — Additional file 1: Supplementary table 1. Odds ratio of CDI in IBD compared to those without IBD using laboratory dataset (Lab) and Hospitalization Admission and Discharge Database (DAD) (A047). [file 12876_2022_2223_MOESM1_ESM.docx]

| Year | Odds Ratio (95% CI) | |
| --- | --- | --- |
|  | Laboratory | A07 (DAD) |
| 2005 | 1.04 (0.49-2.20) | 1.55 (0.78-3.07) |
| 2006 | 1.46 (0.79-2.73) | 1.59 (0.83-3.05) |
| 2007 | 2.51 (1.33-4.73) | 2.29 (1.17-4.45) |
| 2008 | 2.64 (1.35-5.17) | 3.12 (1.69-5.76) |
| 2009 | 1.13 (0.56-2.30) | 1.37 (0.69-2.72) |
| 2010 | 2.56 (1.42-4.59) | 3.08 (1.65-5.77) |
| 2011 | 2.02 (1.08-3.76) | 3.41 (1.90-6.11) |
| 2012 | 1.49 (0.66-3.37) | 1.96 (0.95-4.05) |
| 2013 | 2.25 (1.24-4.07) | 3.12 (1.71-5.68) |

**Supplementary table 1: Odds ratio of CDI in IBD compared to those without IBD using laboratory dataset (Lab) and Hospitalization Admission and Discharge Database (DAD) (A047)**
